# Supplementary material for: HIVprotI: an integrated web based platform for prediction and design of HIV proteins inhibitors
Source: J Cheminform. 2018 Mar 9;10:12. doi: 10.1186/s13321-018-0266-y (PMC5845081; doi:10.1186/s13321-018-0266-y)
Supplement: Supplementary file 2 — Additional file 2. Source code of HIVProtI web server. [file 13321_2018_266_MOESM2_ESM.zip › HIVprotI_Source-code/hivprotI/weka-3-6-6/doc/weka/gui/sql/Messages.html]

Messages


---


|  |  |  |
| --- | --- | --- |
| **Overview** \| **Package** \| **Class** \| **Tree** \| **Deprecated** \| **Index** \| **Help** \| **Weka's home** | |  |
| **PREV CLASS**   **NEXT CLASS** | **FRAMES**    **NO FRAMES**     **All Classes** |  |
| SUMMARY: NESTED \| FIELD \| CONSTR \| METHOD | DETAIL: FIELD \| CONSTR \| METHOD |  |


---


## weka.gui.sql Class Messages

```
java.lang.Object
  weka.gui.sql.Messages
```

---

``` public class Messages extends java.lang.Object ```

Messages.

**Version:**
:   $Revision: 7059 $

**Author:**
:   shingo.yamagami@ksk-sol.jp

---

**Constructor Summary**

`Messages()`


| **Method Summary** | |
| --- | --- |
| `static Messages` | `getInstance()`             getInstance. |
| `static java.lang.String` | `getString(java.lang.String key)`             getString. |
| `static java.lang.String` | `getString(java.lang.String key, java.util.Locale locale)`             getString. |

**Methods inherited from class java.lang.Object**

`equals, getClass, hashCode, notify, notifyAll, toString, wait, wait, wait`

**Constructor Detail**

### Messages

```
public Messages()
```


**Method Detail**

### getInstance

```
public static Messages getInstance()
```

:   getInstance.

    :   **Returns:**

---


### getString

```
public static java.lang.String getString(java.lang.String key)
```

:   getString.

    :   **Parameters:**: `key` - **Returns:**

---


### getString

```
public static java.lang.String getString(java.lang.String key,
                                         java.util.Locale locale)
```

:   getString.

    :   **Parameters:**: `key` -: `locale` - **Returns:**


---


|  |  |  |
| --- | --- | --- |
| **Overview** \| **Package** \| **Class** \| **Tree** \| **Deprecated** \| **Index** \| **Help** \| **Weka's home** | |  |
| **PREV CLASS**   **NEXT CLASS** | **FRAMES**    **NO FRAMES**     **All Classes** |  |
| SUMMARY: NESTED \| FIELD \| CONSTR \| METHOD | DETAIL: FIELD \| CONSTR \| METHOD |  |


---
